# Supplementary material for: Buffering Mitigates Chondrocyte Oxidative Stress, Metabolic Dysfunction, and Death Induced by Normal Saline: Formulation of a Novel Arthroscopic Irrigant
Source: Int J Mol Sci. 2024 Jan 20;25(2):1286. doi: 10.3390/ijms25021286 (PMC10816598; doi:10.3390/ijms25021286)
Supplement: Supplementary file 1 [file ijms-25-01286-s001.zip › ijms-2812231-supplementary.pdf]

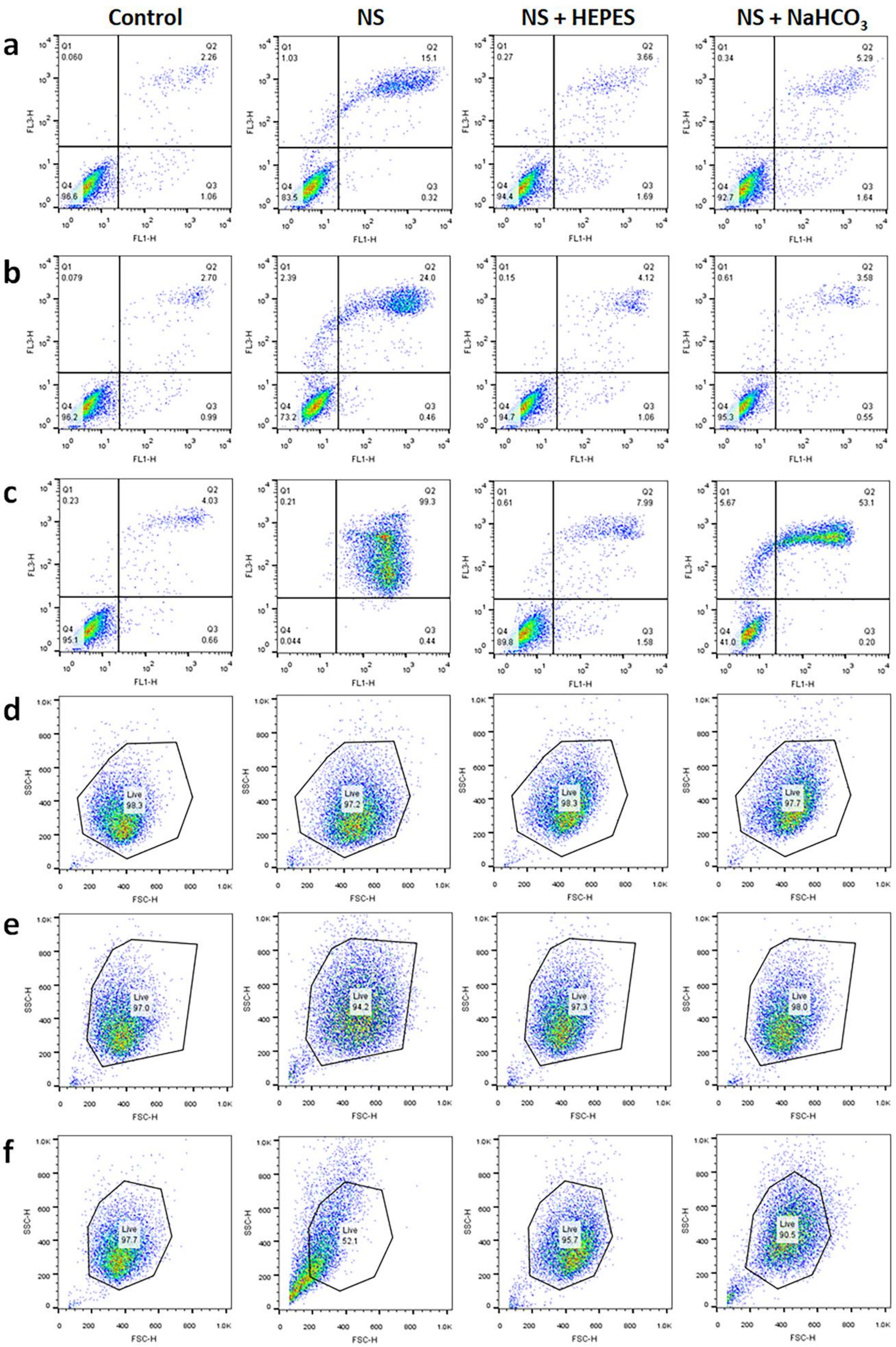
Control
NS
NS + HEPES
NS + $NaHCO_3$
a
Q1 0.060
Q2 2.26
Q4 98.6
Q3 1.06
Q1 1.03
Q2 15.1
Q4 83.5
Q3 0.32
Q1 0.27
Q2 3.66
Q4 94.4
Q3 1.69
Q1 0.34
Q2 5.29
Q4 92.7
Q3 1.64
b
Q1 0.079
Q2 2.70
Q4 96.2
Q3 0.99
Q1 2.39
Q2 24.0
Q4 73.2
Q3 0.46
Q1 0.15
Q2 4.12
Q4 94.7
Q3 1.06
Q1 0.61
Q2 3.58
Q4 95.3
Q3 0.55
c
Q1 0.23
Q2 4.03
Q4 95.1
Q3 0.66
Q1 0.21
Q2 99.3
Q4 0.044
Q3 0.44
Q1 0.61
Q2 7.99
Q4 89.8
Q3 1.58
Q1 5.67
Q2 53.1
Q4 41.0
Q3 0.20
FL3-H
FL1-H
d
Live 98.3
Live 97.2
Live 98.3
Live 97.7
e
Live 97.0
Live 94.2
Live 97.3
Live 98.0
f
Live 97.7
Live 52.1
Live 95.7
Live 90.5
SSC-H
FSC-H

Supplementary Figure S1. Effect of normal saline (NS) and buffers (25 mM HEPES or 0.5 mM sodium bicarbonate) on chondrocyte death using a flow cytometer. (a-c) Representative plots for annexin V (FL1-H; x-axis)/propidium iodide (PI) (FL3-H; y-axis) staining after irrigant incubation for 30 minutes (a), 1 hour (b), and 3 hours (c). Q1: annexin V negative/PI positive, Q2: annexin V positive / PI positive, Q3: annexin V positive / PI negative, Q4: annexin V negative / PI negative. (d-f) Representative FSC vs SSC plots for cell morphology after irrigant incubation for 30 minutes (d), 1 hour (e), and 3 hours (f). FSC-H (forward scatter height; x-axis) and SSC-H (side scatter height; y-axis) represent cell size and granularity, respectively.

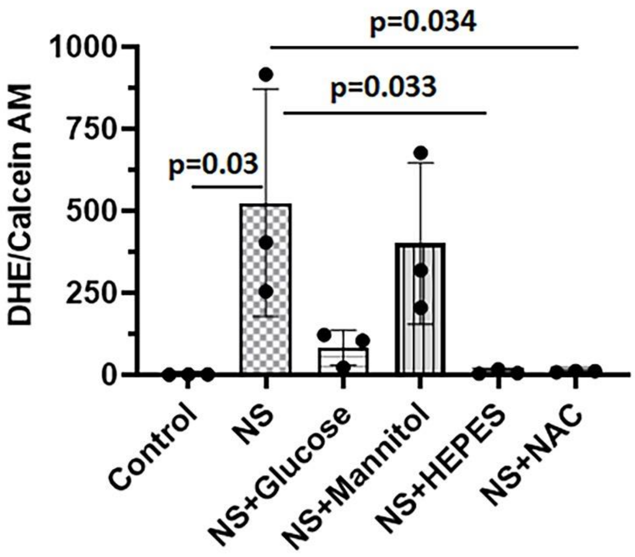

Supplementary Figure S2. Effect of 0.9% normal saline (NS) and various supplements on oxidative stress in monolayer primary bovine chondrocytes. Glucose (Sigma-Aldrich): 31.52 mM, Mannitol (Research Products International): 31.52 mM, HEPES: 25 mM, NAC (Sigma-Aldrich): 2.5 mM with 26 mM sodium bicarbonate (Research Products International) (n = 3).

Supplementary Table S1. List of metabolites showing ≥ 2-fold changes between control and 0.9% normal saline (NS). Chondrocytes were treated with each irrigation solution for 30 minutes (n = 6). Positive values of fold change (up): NS or NS + HEPES > control, negative values of fold change (down): NS or NS + HEPES < control.

	**NS *versus* Control**		**NS + HEPES *versus* Control**	
**Metabolites (up)**	**Fold change**	**p-value**	**Fold change**	**p-value**
Glucose 6-phosphate	70.3	5.9E-09	23.4	1.5E-04
Ribose 5-phosphate	34.3	5.8E-09	6.8	1.2E-02
Fructose 6-phosphate	7.5	5.9E-09	2.8	7.6E-04
O-octanoyl-R-carnitine	5.0	1.1E-07	7.0	6.2E-09
D-Ribulose 5-phosphate	4.2	5.8E-09	2.0	1.6E-05
O-propanoyl-carnitine	4.2	1.9E-08	3.9	6.1E-08
Inosine	3.2	5.8E-09	0.6	4.8E-03
Mannose	3.0	6.3E-08	1.9	6.8E-04
Guanosine	2.9	5.9E-09	1.7	1.2E-04
Adenosine monophosphate (AMP)	2.3	5.8E-09	0.8	9.1E-03
Nicotinamide adenine dinucleotide phosphate (NADP+)	2.2	7.7E-08	1.7	8.1E-05
Deoxyguanosine diphosphate (dGDP)	2.2	5.8E-09	0.6	3.3E-05
O-hexanoyl-R-carnitine	2.1	2.7E-08	2.1	2.3E-08
**Metabolites (down)**	**Fold change**	**p-value**	**Fold change**	**p-value**
Reduced nicotinamide adenine dinucleotide phosphate (NADPH)	-31.1	6.6E-09	-0.8	7.2E-04
Alpha-Ketoglutarate (KG)	-22.0	5.8E-09	-15.5	5.8E-09
Alpha-Ketoisovalerate (KIV)	-12.4	6.1E-09	-4.8	8.3E-09
Cytidine triphosphate (CTP)	-11.8	5.8E-09	-1.4	5.7E-06
Alpha-Keto-beta-Methylvalerate (KMV)	-10.2	5.8E-09	-10.2	5.8E-09
Nicotinamide adenine dinucleotide + hydrogen (NADH)	-9.6	1.2E-07	-0.8	7.0E-03
O-oleoylcarnitine	-9.1	1.2E-05	-1.0	9.8E-01
Alpha-Ketoisocaproate (KIC)	-9.0	5.8E-09	-6.8	5.8E-09
Lactate	-7.2	5.8E-09	-3.3	5.8E-09
Xanthosine	-5.7	0.020	-0.6	4.4E-02
Pyruvic acid	-5.5	5.8E-09	-2.6	6.4E-09
Cytidine diphosphate (CDP)	-4.8	5.8E-09	-2.2	5.8E-09
Oxidized glutathione (GSSG)	-4.8	7.9E-08	-1.7	1.4E-04
O-Phosphoethanolamine	-4.4	7.1E-09	-3.6	9.1E-09

Dihydroxyacetone phosphate (DHAP)	-4.1	5.8E-09	-2.2	5.8E-09
Beta-Hydroxybutyrate (3-Hydroxybutyrate)	-3.8	5.8E-09	-2.3	5.9E-09
Inosine monophosphate (IMP)	-3.8	1.4E-07	-1.8	8.2E-05
Malate	-3.7	1.7E-08	-7.3	6.9E-09
Citrulline	-3.7	5.8E-09	-3.6	5.8E-09
Deoxythymidine triphosphate (dTTP)	-3.6	7.4E-07	-2.4	9.4E-06
Succinic acid	-3.5	8.1E-09	-4.2	6.7E-09
Guanosine triphosphate (GTP)	-3.2	6.0E-09	-0.9	4.5E-01
Hypoxanthine	-3.1	4.8E-07	-1.0	0.838
Fumarate	-3.0	8.3E-08	-3.7	2.7E-08
Glutarylcarnitine	-3.0	5.9E-09	-1.8	2.8E-08
Pyruvate	-2.9	5.8E-09	-2.0	5.9E-09
Adenosine diphosphate (ADP) ribose	-2.9	6.1E-09	-1.6	6.3E-07
Carnitine	-2.9	5.8E-09	-1.4	1.7E-08
Lysine	-2.8	5.8E-09	-2.3	5.8E-09
Malic acid	-2.8	1.8E-06	-4.0	2.3E-07
Fumaric acid	-2.8	2.9E-06	-4.1	3.4E-07
Uridine triphosphate (UTP)	-2.5	5.8E-09	-1.0	0.506
Deoxyadenosine triphosphate (dATP)	-2.4	5.8E-09	-1.6	2.2E-08
Citric acid	-2.4	5.7E-04	-2.5	4.8E-04
Deoxyguanosine triphosphate (dGTP)	-2.1	6.2E-09	-0.9	3.6E-04
